# Nebulized magnesium sulfate reduces postoperative sore throat following general anesthesia: a systematic review, meta-analysis, and trial sequential analysis

**DOI:** 10.1186/s12871-026-04066-6

**Published:** 2026-07-06

**Authors:** Sandara Bashir, Muhammad Mustafa Khan, Muhammad Hamad Khan, Abdul Haseeb, Abdullah Abdullah, Abdul Saboor, Habib Ur Rehman, Rayaan Jan, Aman Ullah, Hassan Khalil, Sayed Shahabuddin, Abdul Muiz, Yahya Samadi, Asiya Shahzad

**Affiliations:** 1 Kabir Medical College, Peshawar, Pakistan; 2 Present Address: Khyber Medical College, Peshawar, Pakistan; 3 Bacha Khan Medical College, Mardan, Pakistan; 4 Department of Curative Medicine, Faculty of Medicine, Kateb University, Kabul, Afghanistan; 5 Peshawar Medical College, Peshawar, Pakistan

**Keywords:** Post-operative sore throat, Magnesium sulfate, Nebulization, Meta-analysis

## Abstract

**Background:**

Post-operative sore throat (POST) frequently complicates general anesthesia involving endotracheal intubation, affecting roughly 60% of individuals and markedly hindering postoperative recovery. The current systematic review and meta-analysis assessed both the safety profile and prophylactic efficacy of preoperative magnesium sulfate administered via nebulization against POST.

**Methods:**

A systematic review and meta-analysis of randomized controlled trials (RCTs) comparing preoperative nebulized magnesium sulfate with placebo or normal saline for POST prevention in adults undergoing elective surgery under general anesthesia was conducted. The primary outcome was POST incidence at 24 h. Secondary outcomes included POST at 2, 4, 8, and 12 h, hoarseness, cough, and hemodynamic adverse events. PROSPERO registration: CRD420261308633.

**Results:**

Twelve RCTs comprising 1,023 patients were included. Nebulized Magnesium Sulfate significantly reduced POST at 24 h compared to control (RR = 0.25; 95% CI: 0.14–0.42; *p* < 0.00001; I² = 62%). Sensitivity analysis excluding an outlier reduced heterogeneity (I² = 7%) while maintaining significance. Significant reductions were also observed at 2 h (RR = 0.43; 95% CI: 0.32–0.58), 4 h (RR = 0.38; 95% CI: 0.19–0.77), and 12 h (RR = 0.34; 95% CI: 0.14–0.79). Subgroup analysis demonstrated comparable efficacy between the 225 mg dose and higher doses, with no statistically significant difference observed. No significant reduction was observed for hoarseness (RR = 0.44; 95% CI: 0.17–1.18) or cough (RR = 0.39; 95% CI: 0.15–1.04), and no increased hemodynamic risk was detected. Trial sequential analysis confirmed robust evidence for POST prevention.

**Conclusion:**

The administration of preoperative nebulized magnesium sulfate serves as an effective and safe measure to decrease POST incidence among adult patients, thereby justifying its inclusion in standard perioperative management protocols.

**Supplementary Information:**

The online version contains supplementary material available at https://doi.org/10.1186/s12871-026-04066-6.

## Introduction

Following general anesthesia that involves endotracheal intubation, adult patients frequently encounter post-operative sore throat (POST). The existing literature indicates that the incidence of this specific adverse airway event can reach up to 60% [1–3]. Although the complication resolves on its own, it continues to contribute significantly to reduced postoperative patient comfort, satisfaction, and impaired quality of recovery in the early postoperative period [4–6]. Therefore, prevention of POST is a relevant and meaningful objective in improving the patient-reported outcomes via perioperative care.

The etiology of POST is multifactorial, yet it primarily stems from inflammatory and mechanical trauma inflicted upon the laryngeal and pharyngeal mucosa during laryngoscopy, tube insertion, and the subsequent inflation of the cuff [7–9]. Factors like endotracheal tube size, cuff pressure, duration of intubation, and repeated airway manipulation further contribute to POST [2, 8, 9]. Multiple treatment modalities have been tested for preventing POST. These include topical local anaesthetics, corticosteroids, ketamine, and non-steroidal anti-inflammatory agents; however, their efficacy has been inconsistent with some associated with undesirable adverse effects [2, 3].

Magnesium Sulfate has recently emerged as a potent agent for preventing POST because of its analgesic and anti-inflammatory properties. It acts primarily via N-methyl-D-aspartate receptor antagonism and inhibition of calcium-dependent nociceptive transmission. Topical administration of magnesium sulfate, mainly via nebulization, causes localized effects on the airway mucosa while minimizing systemic absorption and hemodynamic instability [10–12]. Multiple randomized controlled trials have analysed the role of topical magnesium sulfate for the prevention of POST in adult patients undergoing elective surgery under general anaesthesia. Most of these studies concluded that there was a reduction in the incidence and severity of POST compared to placebo or normal saline [13].

Furthermore, multiple randomized controlled trials have suggested that Magnesium Sulfate reduces POST, these individual studies have been limited by their small sample sizes and variable dosing regimens. Although, previous meta-analyses, such as the comprehensive review by Singh et al. [14], have demonstrated the prophylactic value of magnesium sulfate, their analysis pooled highly heterogeneous ‘topical’ administration routes, specifically grouping oropharyngeal gargles and lozenges together with aerosolized nebulization. This methodological grouping introduces significant variability, as gargles primarily coat the upper oropharynx, whereas nebulization provides targeted inhalational delivery to the glottic and tracheal mucosa where endotracheal cuff trauma actually occurs. Consequently, there remains a distinct knowledge gap regarding the isolated efficacy of the inhalational route. This updated meta-analysis addresses this gap by strictly evaluating preoperative nebulized Magnesium Sulfate against nebulized Normal Saline or a matching inert placebo in preventing postoperative sore throat in adult patients undergoing general anaesthesia with endotracheal intubation.

### Methodology

#### Protocol registration

The present systematic review conformed to the principles laid out by Cochrane Collaboration [15] and the Preferred Reporting Items for Systematic Revews and Meta-Analysis (PRISMA) statement [16]. This included all aspects from designing, implementation, analysis and results presentation of the current research work. Moreover, the protocol of this study had been pre-registered at the International Prospective Register of Systematic Reviews (PROSPERO). registration no (CRD420261308633).

#### Search strategy

An electronic search of PubMed, Embase and Scopus was conducted from inception till February 2026 without any language restrictions. The following search strategy was used:

- (“Magnesium Sulfate” OR “Magnesium Sulfate” OR “MgSO4” OR “Magnesium”)
- AND (“Nebulizer” OR “Nebulizers” OR “Nebulization” OR “Nebulisation” OR “Inhalation” OR “Aerosol” OR “Aerosols” OR “Atomization” OR “Atomisation”)
- AND (“Postoperative Sore Throat” OR “Sore Throat” OR “Hoarseness” OR “Cough” OR “Pharyngitis” OR “Intubation” OR “Endotracheal Intubation”). The boolean operators (AND/OR) were used to optimize the search strategy so that all the relevant research could be obtained. Furthermore, manual searching was also done on the citation lists obtained from trials as well as previous meta-analyses, and other articles related to our topic were read to find more relevant articles. Articles such as systematic/narrative review, poster/conference abstract, case report and letter to editor which did not qualify for inclusion criteria were excluded. The complete search strategy is detailed in Supplementary Table 2.

#### Eligibility criteria and study selection

Articles were considered suitable for inclusion based on the following parameters:

(i) Study Design: Randomized controlled trials (RCTs) in any language.
(ii) Population: Adult patients (aged > 18 years) classified as American Society of Anesthesiologists (ASA) physical status I or II, undergoing elective surgeries requiring general anaesthesia with endotracheal intubation.
(iii) Intervention: Pre-operative administration of nebulized magnesium sulfate (any dose) prior to induction of anaesthesia.
(iv) Comparator: Nebulized normal saline (0.9%) or a matching placebo.
(v) Outcomes: Assessed the incidence or severity of Post-Operative Sore Throat (POST) at any post-operative time point.

Conversely, trials were excluded based on these criteria: (i) pediatric cohorts; (ii) individuals presenting with a recent upper respiratory tract infection (URI) or an existing sore throat; (iii) anticipated difficult airways necessitating more than one intubation attempt; (iv) interventions utilizing gargled or intravenous magnesium sulfate; or (v) an absence of retrievable data regarding the primary outcome.

Following the initial literature search, retrieved citations were uploaded to the Rayyan AI web application, which facilitated the identification and removal of duplicate records. Subsequently, two independent investigators (AA & MHK) evaluated the remaining records to select studies meeting the predefined inclusion criteria. This evaluation commenced with a review of abstracts and titles, followed by a comprehensive full-text appraisal to confirm eligibility. Any discordance between the reviewers was resolved through consultation with the senior investigator (MMK). Ultimately, twelve randomized controlled trials satisfied the inclusion parameters and were incorporated into the final analysis.

#### Data extraction and outcomes

Data extraction was done systematically to enhance reliability, consistency and pertinence of the findings. Three reviewers extracted the data using a standard Excel spreadsheet. Differences that occurred during the process were sorted out through discussions; if not possible, referral to the senior author was sought. The above method was adopted to enable proper data extraction for analysis. Outcome measures: The outcomes included POST occurrence at 24 h after extubation as a primary outcome measure. Secondary outcomes included the time course of POST occurrence at 2, 4, 8 and 12 h respectively, incidence of postoperative hoarsness, incidence of postoperative cough and hemodynamic adverse effects like hypotension and bradycardia.

#### Bias assessment

The Cochrane RoB 2 instrument [17, 18] was utilized to evaluate the risk of bias across the included trials. This instrument assesses potential bias across five distinct domains: (1) the randomization procedure; (2) departures from the assigned interventions; (3) incomplete outcome reporting; (4) outcome measurement methodologies; and (5) the selective reporting of results.

#### GRADE assessment

Quality of evidence for inclusion in this meta-analysis was assessed independently by two reviewers (AS and MHK) using the GRADE tool through the GRADEpro Guideline Development Tool [19]. Evidence quality was graded according to four different levels: High, Moderate, Low, and Very Low [20]. Discrepancies between reviewers were sorted out through consensus.

### Statistical analysis

To ensure computational precision, all quantitative analyses were executed using Review Manager (RevMan) software, Version 5.4.1. Binary outcomes were evaluated via the Mantel-Haenszel technique and are reported as risk ratios (RRs) alongside their corresponding 95% confidence intervals (CIs). A random-effects meta-analytical framework was applied to account for variance across the included studies, with the findings visually depicted in forest plots. Inter-study heterogeneity was quantified utilizing Cochran’s Q statistic and the I^2^ index. An I^2^ value of ≥ 50% indicated moderate heterogeneity, while values between 75% and 100% denoted substantial heterogeneity. Furthermore, Potential publication bias for the primary outcome was assessed visually through the generation of a funnel plot to evaluate for small-study effects and distributional symmetry.

Trial Sequential Analysis (TSA) was conducted using TSA software version 0.9.5.10 beta (Copenhagen Trial Unit, Centre for Clinical Intervention Research, Rigshospitalet, Copenhagen, Denmark, www.ctu.dk/tsa). The required optimal information size (OIS) was estimated setting the Type I error (alpha) boundary at 0.05 (two-sided) and the Type II error (beta) boundary at 0.20, achieving 80% statistical power. The anticipated relative risk reduction (RRR) was prespecified as 50%. This threshold was selected to be substantially more conservative than the 78% RRR observed in prior external evidence [14], thereby avoiding circular reliance on the observed pooled effect of the current meta-analysis, which could result in an unrealistically optimistic effect assumption and a consequently underestimated DARIS [21]. The pooled control event proportion was determined empirically from the baseline incidence observed in the control arms of the included trials. To account for clinical diversity across the studies, we applied a model variance-based heterogeneity correction (D^2^). Trial sequential monitoring boundaries for benefit, harm, and futility were constructed using the O’Brien-Fleming alpha-spending function.

## Results

### Search results

**Fig. 1:**
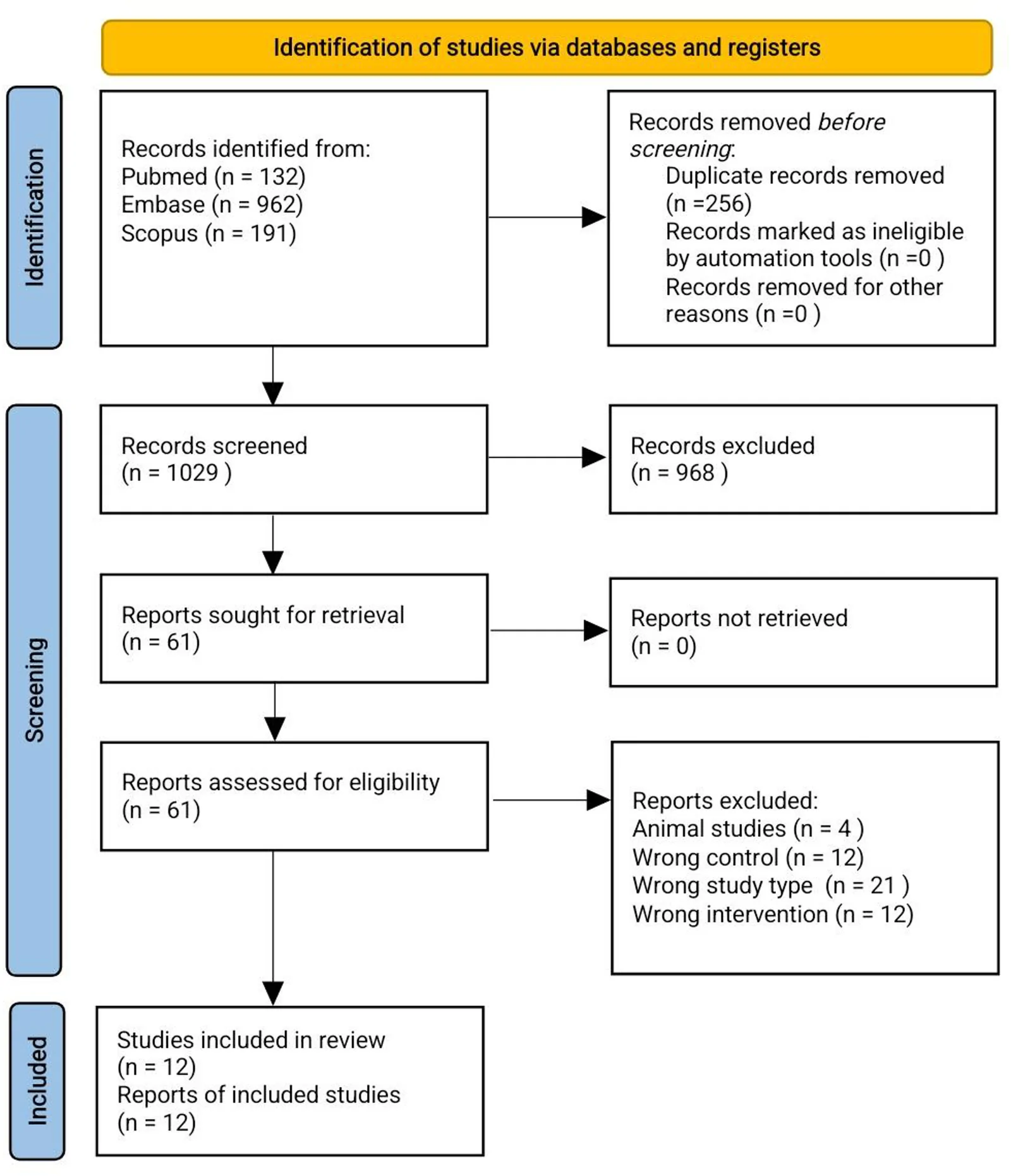
Prisma flow chart

A total of 1,285 records were identified through electronic database searches, including PubMed (*n* = 132), Embase (*n* = 962), and Scopus (*n* = 191). After removing 256 duplicates, 1,029 records were screened for relevance. Following title and abstract screening, 968 records were excluded. The full texts of the remaining 61 articles were retrieved and assessed for eligibility. Of these, 49 studies were excluded for the following reasons: wrong study design (*n* = 21), wrong intervention (*n* = 12), wrong control group (*n* = 12), and animal studies (*n* = 4).Ultimately, 12 randomized controlled trials met the inclusion criteria and were included in the final systematic review and meta-analysis. The study selection process is illustrated in Fig. 1.

### Study characteristics results

The quantitative synthesis included 12 Randomized Controlled Trials (RCTs) published between 2016 and 2026 [11, 12, 22–31].The studies were conducted across multiple countries, including India (*n* = 8), Egypt (*n* = 2), Iraq (*n* = 1), and Nigeria (*n* = 1). The pooled population comprised a total of 1,023 patients, with 519 patients assigned to the nebulized magnesium sulfate group and 504 patients to the normal saline (control) group. All included studies recruited adult patients classified as ASA physical status I or II undergoing elective surgery under general anaesthesia with endotracheal intubation. The intervention protocols utilized nebulized magnesium sulfate at varying doses, ranging from 150 mg to 500 mg, administered pre-operatively. The details of the study designs and patient characteristics are summarized in Tables 1 and 2.

**Table 1:** Study characteristics

Study ID	Study Type	Country	ASA (I/II)	Surgery Type	Intervention	Control	Total Sample	Intervention (*n*)	Control (*n*)	MgSO4 Dose (mg)	Neb Vol (mL)	Duration (min)	POST at 24 h (M vs. S)	Other Outcomes
Al-awwady, 2025 [22]	Prospective Single Blind RCT	Iraq	I & II	Thyroidectomy	MgSO4	Normal Saline	40	20	20	250	2.5	15	2/20 vs. 10/20	POST 0–24 h, Severity, Age, BMI
Brindha et al., 2020 [23]	Prospective Longitudinal RCT	India	I & II	Elective	MgSO4	Normal Saline	100	50	50	150	3	15	0/50 vs. 35/50	POST 0–24 h, Surgery Duration, Age, Weight, Hypotension
Jain & Barasker, 2017 [24]	Prospective Double Blind RCT	India	I & II	Laparoscopic (Cholecystectomy)	MgSO4	Normal Saline	100	50	50	225	3	10	2/50 vs. 7/50	POST 0–24 h, Severity, HR, BP
Kamel & Amin, 2020 [27]	Prospective Double Blind RCT	Egypt	I & II	Elective (Abdominal/Limb)	MgSO4	Normal Saline	52	26	26	250	5	15	7/26 vs. 24/26	POST 0–24 h, Hoarseness, Surgery Duration, Age, BMI
Mitra et al., 2024 [25]	Prospective Double Blind RCT	India	I & II	Elective Laparoscopic	MgSO4	Normal Saline	100	50	50	250	3	15	25/50 vs. 50/50	POST 0–48 h, Hoarseness, Cough
Orji et al., 2020 [29]	Prospective Double Blind RCT	Nigeria	I & II	Elective (Supine)	MgSO4	Normal Saline	66	33	33	250	5	10	0/33 vs. 8/33	POST 0–24 h, Severity, Surgery Duration, Age
Padma et al., 2020 [28]	Prospective Double Blind RCT	India	I & II	Elective	MgSO4	Normal Saline	100	50	50	225	3	15	2/50 vs. 9/50	POST 0–24 h, Severity, Age, Smoking
Rajan et al., 2017 [12]	Prospective Double Blind RCT	India	I & II	Elective (Abdominal/Limb)	MgSO4	Normal Saline	45	30	15	250 + 500	5	15	1/30 vs. 6/15	POST 0–24 h, Hoarseness, Cough, HR, Arterial Pressure
Sharma et al., 2017[31]	Prospective Double Blind RCT	India	I & II	Lumbar Spine Surgery (Prone)	MgSO4	Normal Saline	140	70	70	225	5	15	5/70 vs. 18/70	POST 1–24 h, Severity, Surgery Duration
Supriya et al., 2026 [26]	Prospective Triple Blind RCT	India	I & II	Elective (General Surgery)	MgSO4	Normal Saline	60	30	30	250	5	15	2/30 vs. 6/30	POST 2–48 h, Hoarseness, Cough, BMI, Extubation Score
Yadav et al., 2016 [11]	Prospective Double Blind RCT	India	I & II	Elective (General Surgery)	MgSO4	Normal Saline	100	50	50	225	3	15	1/50 vs. 9/50	POST 0–24 h, Severity, Age, Weight
Wali et al., 2025 [30]	Prospective Double Blind RCT	India & Bengal	I & II	Elective	MgSO4	Normal Saline	120	60	60	225	5	15	4/60 vs. 9/60	POST 1–24 h, Severity, Hoarseness, Cough, BMI, Surgery Duration

Trial sample sizes reflect only the specific intervention (nebulized MgSO4) and control (saline/placebo) arms extracted for this meta-analysis; third-arm comparators from the original studies were excluded

**Table 2:** Patient characteristics

study id	mean age (years)		gender male/female (*n*)		BMI (kg/m2)		weight		Duration of Surgery (min.)		ASA Status I/II	
	Intervention	control	Intervention	control	Intervention	control	Intervention	control	Intervention	control	Intervention	control
Al-awwady, 2025 [22]	47.65 ± 7.52	45.85 ± 9.7	8/12	8/12	26.2 ± 2.29	24.5 ± 2.27	NR	NR	NR	NR	13/7	14/6
Brindha et al., 2020 [23]	37.2 ± 11.7	37.06 ± 11.4	22/28	27/23	NR	NR	NR	NR	NR	NR	NR	NR
Jain & Barasker, 2017 [24]	NR	NR	NR	NR	NR	NR	NR	NR	NR	NR	NR	NR
Kamel & Amin, 2020 [27]	30.8 ± 8.3	31.5 ± 6.9	14/12	14/12	29.4 ± 1.8	29.7 ± 2.9	NR	NR	119.9 ± 21	121.7 ± 23	NR	NR
Mitra et al., 2024 [25]	38.80 ± 8.33	39.28 ± 8.91	NR	NR	NR	NR	64.80 ± 8.40	67.71 ± 9.24	NR	NR	NR	NR
Orji et al., 2020 [29]	44.6 ± 13.3	41.2 ± 13.3	20/13	14/19	NR	NR	68.6 ± 8.9	66.6 ± 9.3	NR	NR	NR	NR
Padma et al. 2020 [28]	42.82 ± 9.106	46.58 ± 9.908	26/24	28/22	NR	NR	63.44 ± 6.478	60.82 ± 6.703	NR	NR	NR	NR
Rajan et al. 2017 [12]	NR	NR	NR	NR	NR	NR	NR	NR	NR	NR	NR	NR
Sharma et al. 2017[31]	37.9 ± 12.37	39.9 ± 12.29	47/23	51/19	NR	NR	67.01 ± 7.73	68.11 ± 7.1	131.43 ± 8.28	127.84 ± 9.71	65/5	63/7
Supriya et al. 2026 [26]	45.03 ± 11.56	46.70 ± 11.1	5/25	5/25	23.25 ± 0.98	22.76 ± 0.92	NR	NR	NR	NR	NR	NR
Yadav et al. 2016 [11]	42.3 ± 10.0	39.4 ± 10.7	26/24	27/23	NR	NR	60.06 ± 2.73	59.22 ± 2.492	NR	NR	NR	NR
Wali et al. 2025 [30]	38.5 ± 10.4	39.1 ± 9.8	28/32	26/34	24.6 ± 3.1	25.1 ± 3.5	NR	NR	112 ± 34	115 ± 37	65 / 35	63 / 37

### Quality assessment results

Seven out of twelve studies were assessed as having a low overall risk of bias, indicating strong methodological rigor. The remaining five studies raised some concerns, suggesting minor limitations primarily in areas such as bias arising from the randomization process or bias in the selection of the reported result. No studies were classified as high risk in our analysis. Importantly, all included studies demonstrated sufficient quality, indicating a generally reliable body of evidence for inclusion in this meta-analysis. A detailed assessment of the risk of bias for each study is provided in Supplementary Fig. 1.

### Certainty of evidence

The GRADE approach, using the GRADEpro Guideline Development Tool, was employed to assess the certainty of evidence. The overall certainty of evidence ranged from high to low across the evaluated outcomes. Evidence for the incidence of POST at 2 h was rated as high quality. The outcomes for POST at 4 and 12 h, as well as the incidence of postoperative cough at 4 h, were assessed as moderate quality, primarily due to serious inconsistency. Conversely, the evidence for POST at 8 and 24 h, hoarseness at 4 h, and hemodynamic instability was graded as low quality, largely driven by a combination of serious inconsistency and imprecision. A detailed assessment is shown in Supplementary Table 1.

### Subgroup analysis

Subgroup analyses were conducted to evaluate potential dose-dependent effects of the intervention. The included trials were stratified into two predefined categories based on the administered dose of nebulized magnesium sulfate: a 225 mg group and a 250–500 mg group. One study was excluded from these specific analyses because its dosage regimen did not conform to either category [23].

### Meta analysis of primary outcome

#### Incidence of post-operative sore throat (POST) at 24 hours

**Fig. 2:**
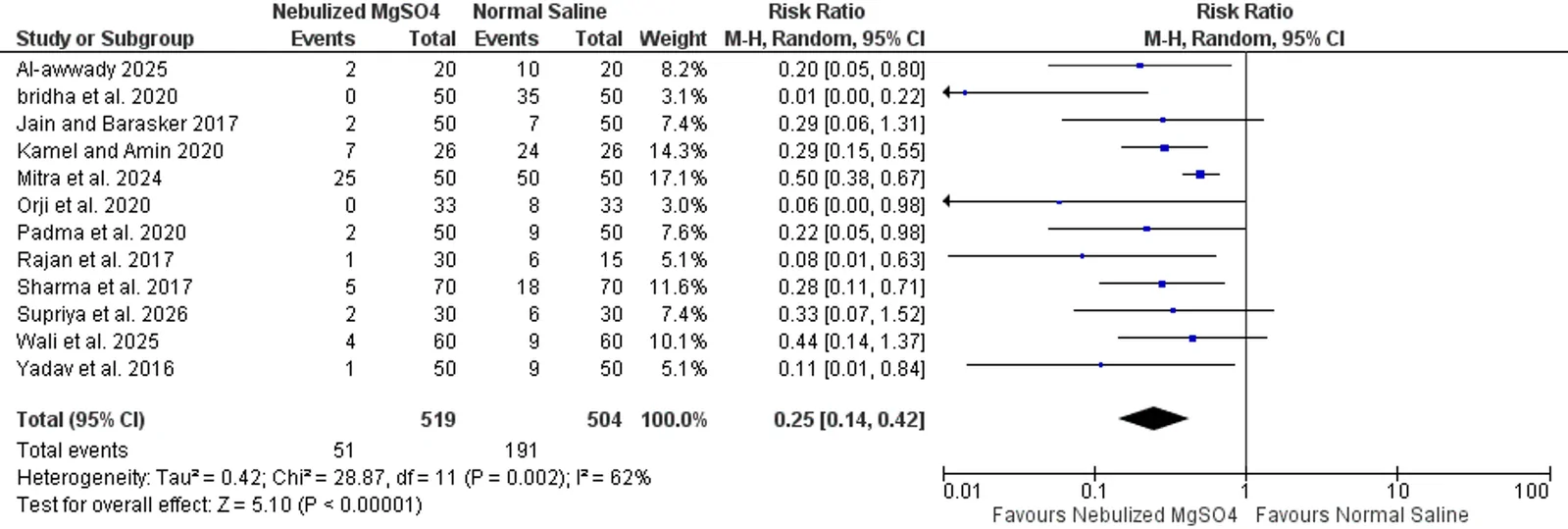
Post-operative sore throat (POST) at 24 hours

**Fig. 3:**
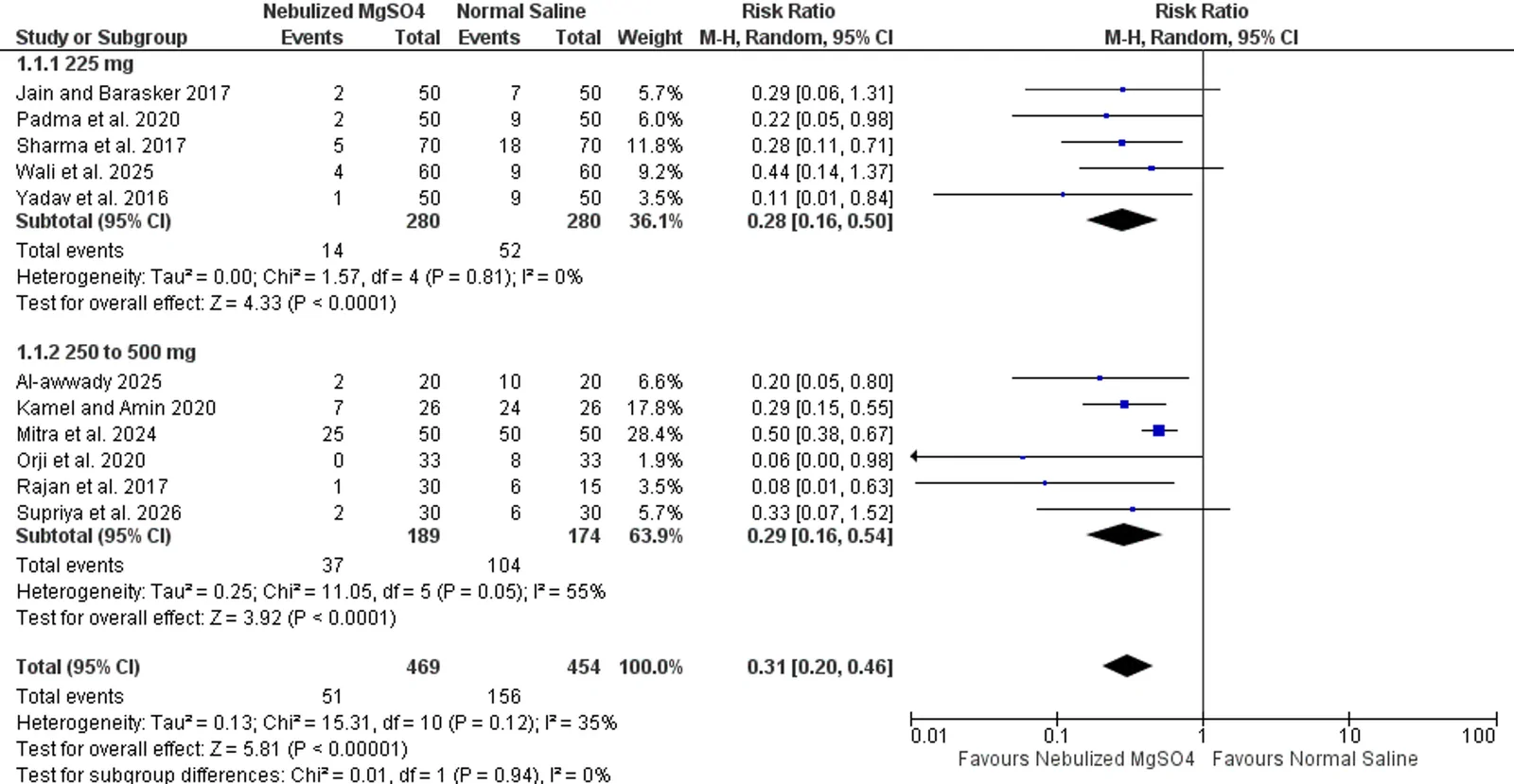
Post-operative sore throat (POST) at 24 hours (subgroup)

The incidence of post-operative sore throat (POST) at 24 h was evaluated in twelve studies, including 1,023 patients (519 in the Nebulized MgSO₄ group and 504 in the normal saline group). The pooled analysis demonstrated that Nebulized MgSO₄ significantly reduced the incidence of POST at 24 h compared with normal saline (RR = 0.25, 95% CI: 0.14–0.42; *p* < 0.00001; I² = 62%) (Fig. 2).To address the observed heterogeneity, a sensitivity analysis was performed by excluding Mitra et al. (2024). The removal of this study significantly reduced heterogeneity from I² = 62% to I² = 7%, with a pooled RR of 0.24 (95% CI: 0.16–0.36; *p* < 0.00001). The detailed results of the sensitivity analysis are presented in Supplementary Fig. 2.Subgroup analysis of the remaining 11 studies demonstrated a significant reduction in risk across both the 225 mg subgroup (RR = 0.28, 95% CI: 0.16–0.50; *p* < 0.0001) and the 250–500 mg subgroup (RR = 0.29, 95% CI: 0.16–0.54; *p* < 0.0001), with no significant difference observed between the subgroups (Fig. 3).

### Meta analysis of secondary outcomes

#### POST at 2 hours (early)

**Fig. 4:**
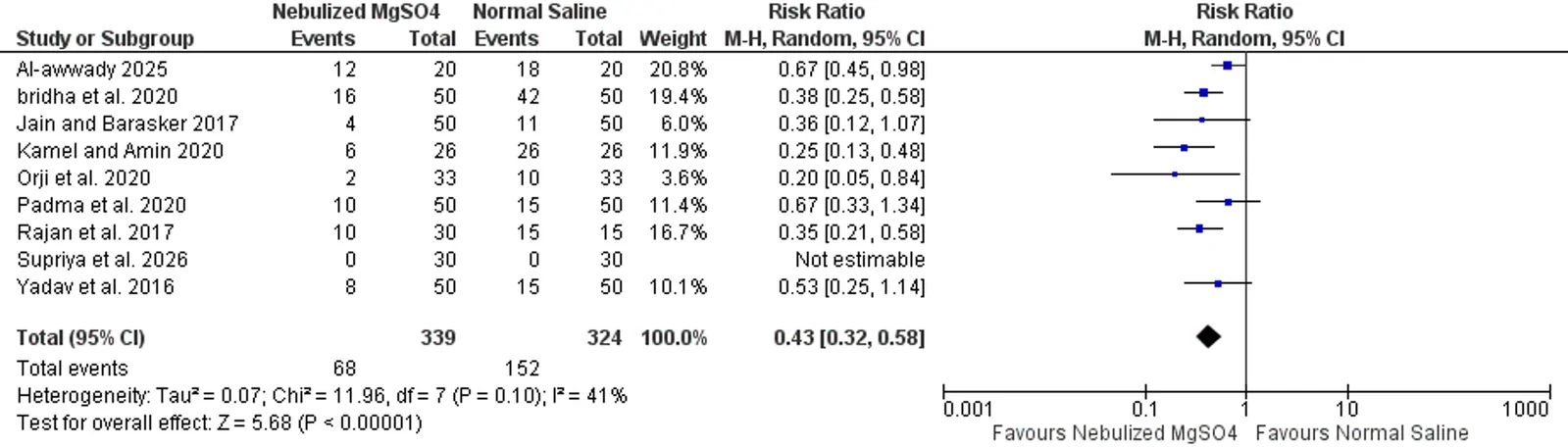
Post-operative sore throat (POST) at 2 hours

**Fig. 5:**
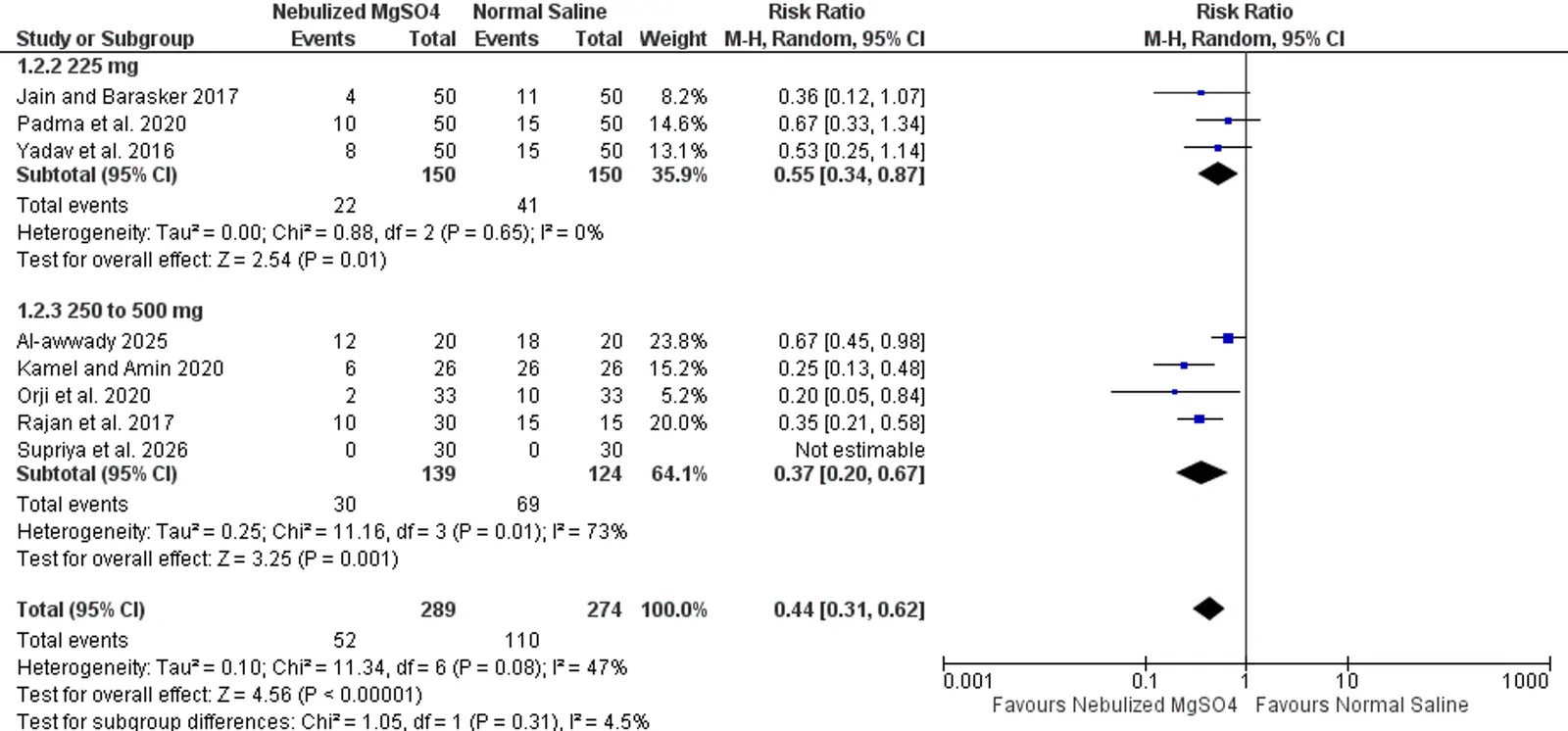
Post-operative sore throat (POST) at 2 hours (subgroup)

Nine studies comprising 663 patients (339 in the Nebulized MgSO₄ group and 324 in the normal saline group) evaluated the incidence of post-operative sore throat (POST) at 2 h. The pooled analysis demonstrated a significantly reduced incidence of POST at 2 h in patients receiving Nebulized MgSO₄ compared with normal saline (RR = 0.43, 95% CI: 0.32–0.58; *P* < 0.00001; I² = 41%) (Fig. 4). Subgroup analysis (*n* = 563 because two studies were excluded) showed a significant reduction for the 225 mg dose (RR = 0.55, 95% CI 0.34– 0.87) and for the 250 to 500 mg dose (RR = 0.37, 95% CI 0.20– 0.67). However, there was no significant difference between these two dosage subgroups (*p* = 0.31,I² = 4.5%) (Fig. 5).

#### POST at 4 hours (early)

**Fig. 6:**
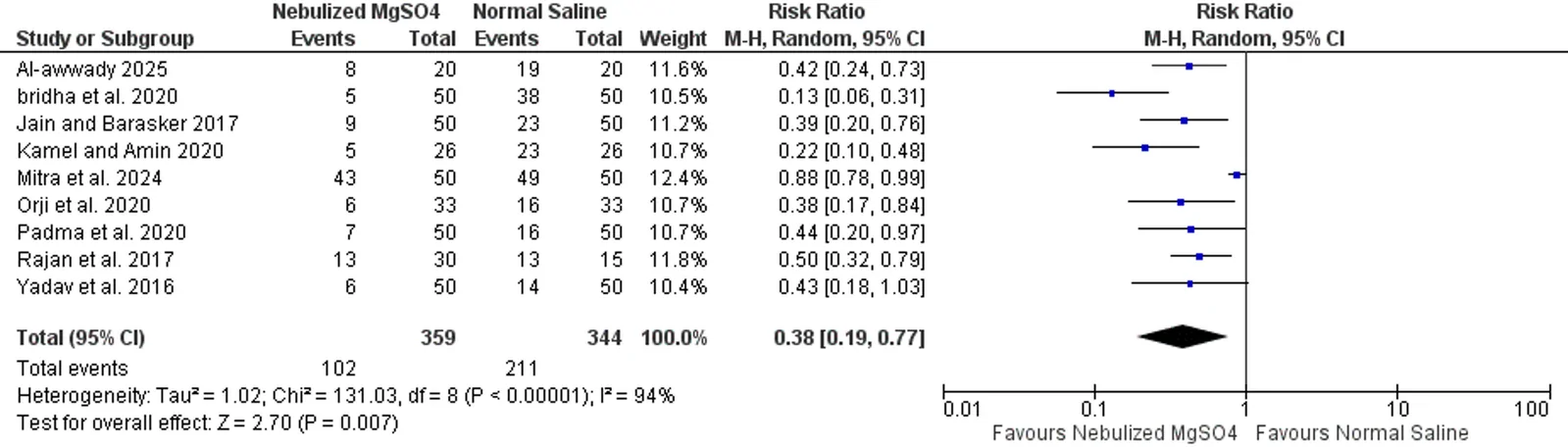
Post-operative sore throat (POST) at 4 hours

**Fig. 7:**
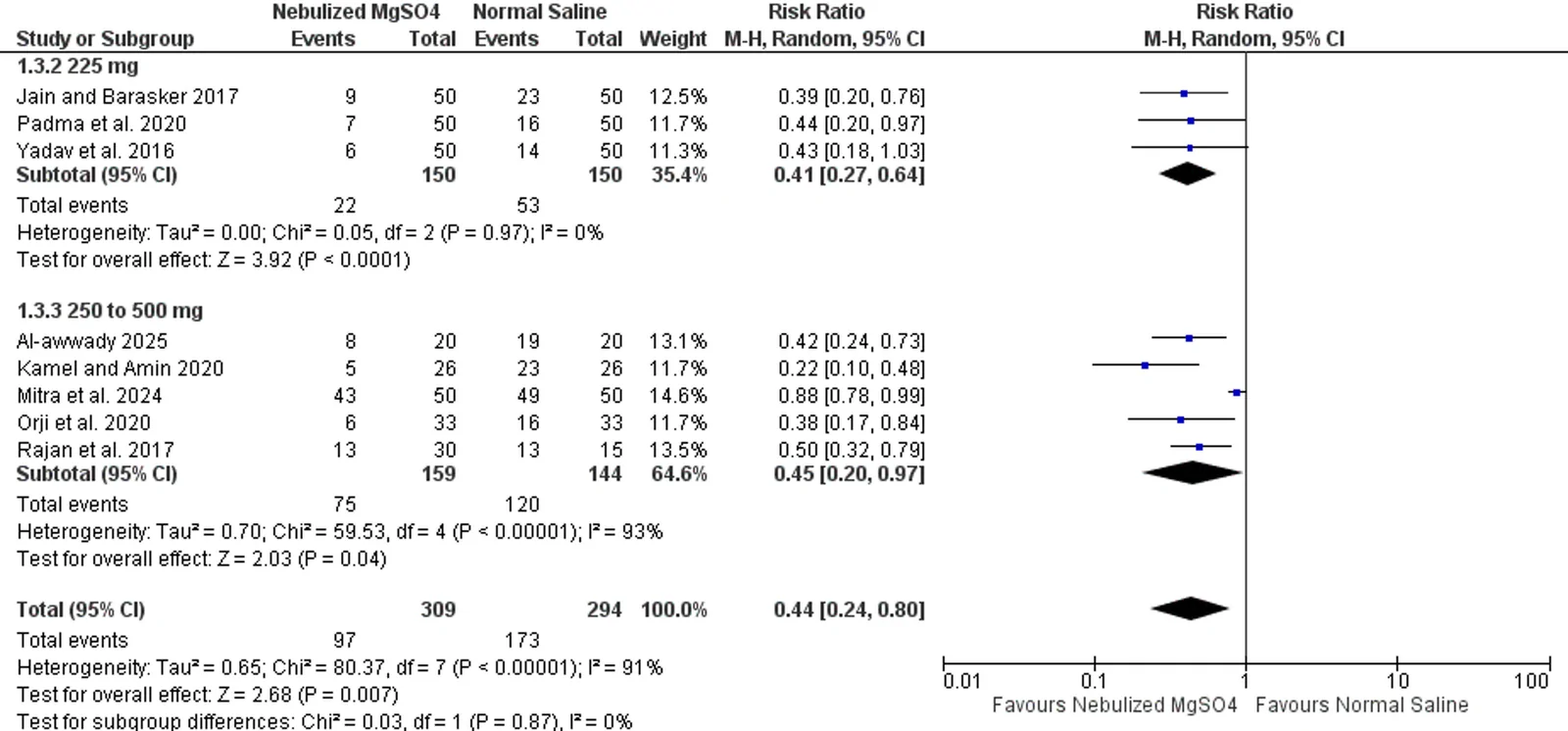
Post-operative sore throat (POST) at 4 hours (subgroup)

Nine studies comprising 703 patients (359 in the Nebulized MgSO₄ group and 344 in the normal saline group) investigated for POST at 4 h. The pooled analysis revealed significantly reduced incidence of POST at 4 h in patients receiving MgSO4 compared with normal saline (RR 0.38, 95% CI 0.19–0.77 ;*P* < 0.007; I²=94% ) (Fig. 6). To address the heterogeneity, a sensitivity analysis was performed excluding Mitra et al. 2024. After exclusion, heterogeneity dropped substantially (I² = 35%), while the result remained significant (RR = 0.36, 95% CI 0.26–0.49, *p* < 0.00001) (Supplementary Fig. 5). Subgroup analysis based on 8 studies (*n* = 603), the dosage of Nebulized MgSO₄, 225 mg Group (RR 0.41, 95% CI 0.27–0.64 ;*P* < 0.0001; I²=0%) and 250 to 500 mg Group (RR 0.45, 95% CI 0.20–0.97 ;*P* < 0.04; I²=93%) revealed no significant difference between the two dosage groups suggesting the treatment effect is consistent across these dose ranges (Fig. 7).

#### POST at 8 hours (early)

**Fig. 8:**
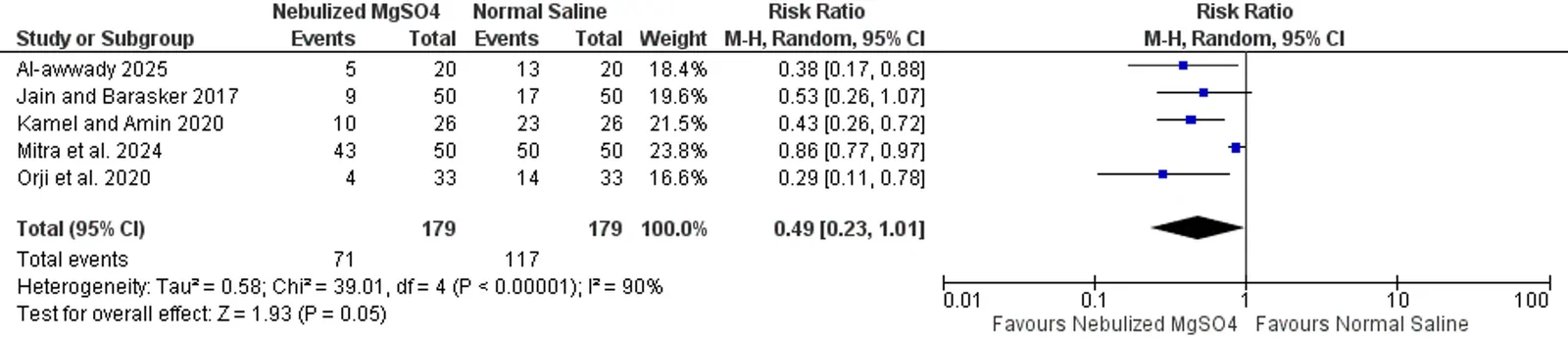
Post-operative sore throat (POST) at 8 hours

**Fig. 9:**
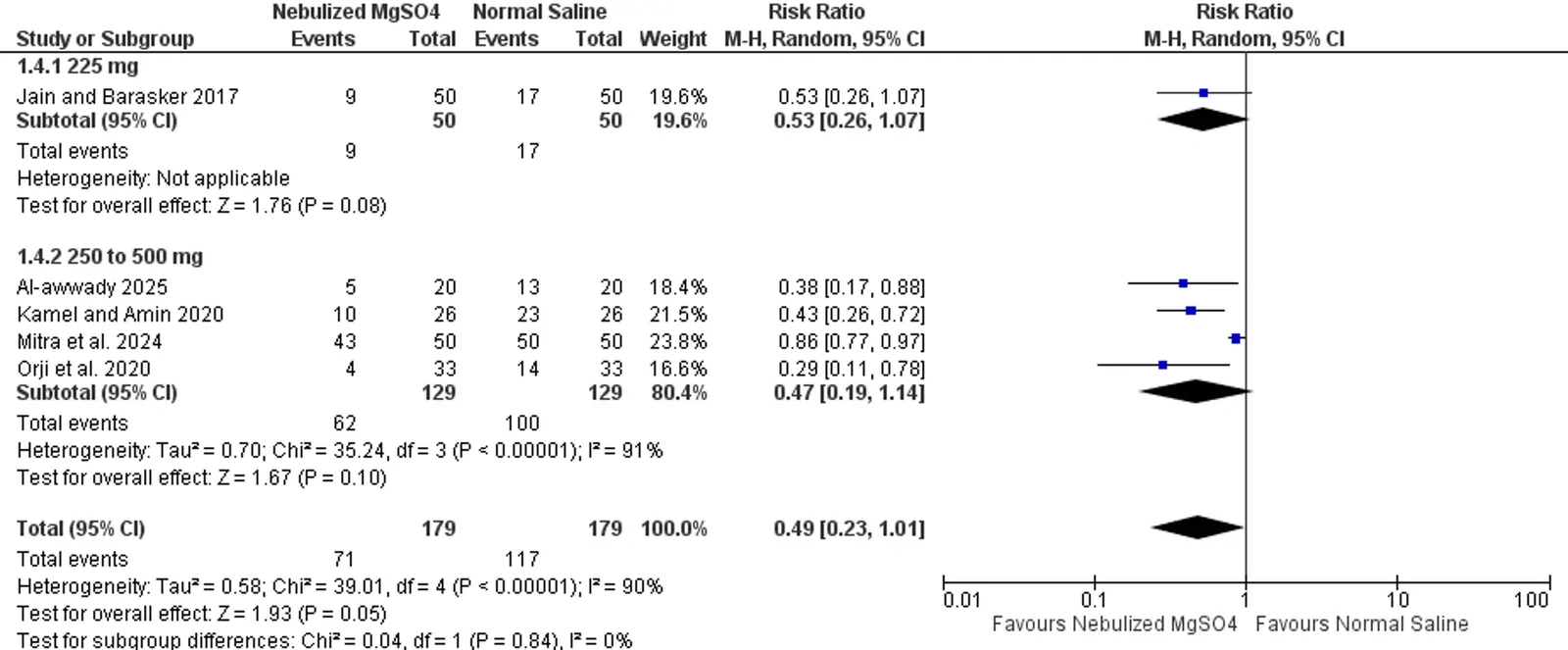
Post-operative sore throat (POST) at 8 hours (subgroup)

Five studies comprising 358 patients (179 in the Nebulized MgSO₄ group and 179 in the normal saline group) investigated for POST at 8 h. The pooled analysis showed a trend toward reduction in incidence of POST at 8 h in patients receiving MgSO4 compared with normal saline (RR 0.49, 95% CI 0.23–1.01 ;*P* = 0.05; I²=90% ) (Fig. 8). To address the heterogeneity, a sensitivity analysis was performed excluding Mitra et al. 2024. After exclusion, heterogeneity dropped substantially (I² = 0%), while the result remained significant (RR = 0.42, 95% CI 0.30– 0.60, *p* < 0.00001). The detailed results of the sensitivity analysis are presented in Supplementary Fig. 4. Subgroup analysis showed 225 mg Group(RR 0.53, 95% CI 0.26–1.07 ;*P* = 0.08) and 250 to 500 mg Group (RR 0.47, 95% CI 0.19–1.14 ;*P* = 0.10; I²=91%), revealed no significant difference in effect (Fig. 9).

#### POST at 12 hours (early)

**Fig. 10:**
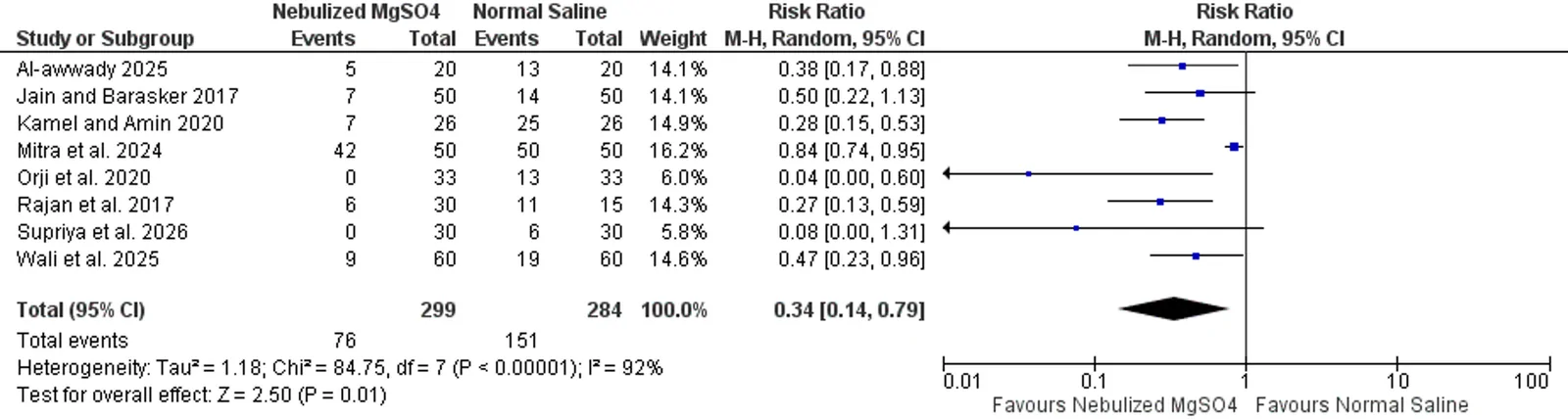
Post-operative sore throat (POST) at 12 hours

**Fig. 11:**
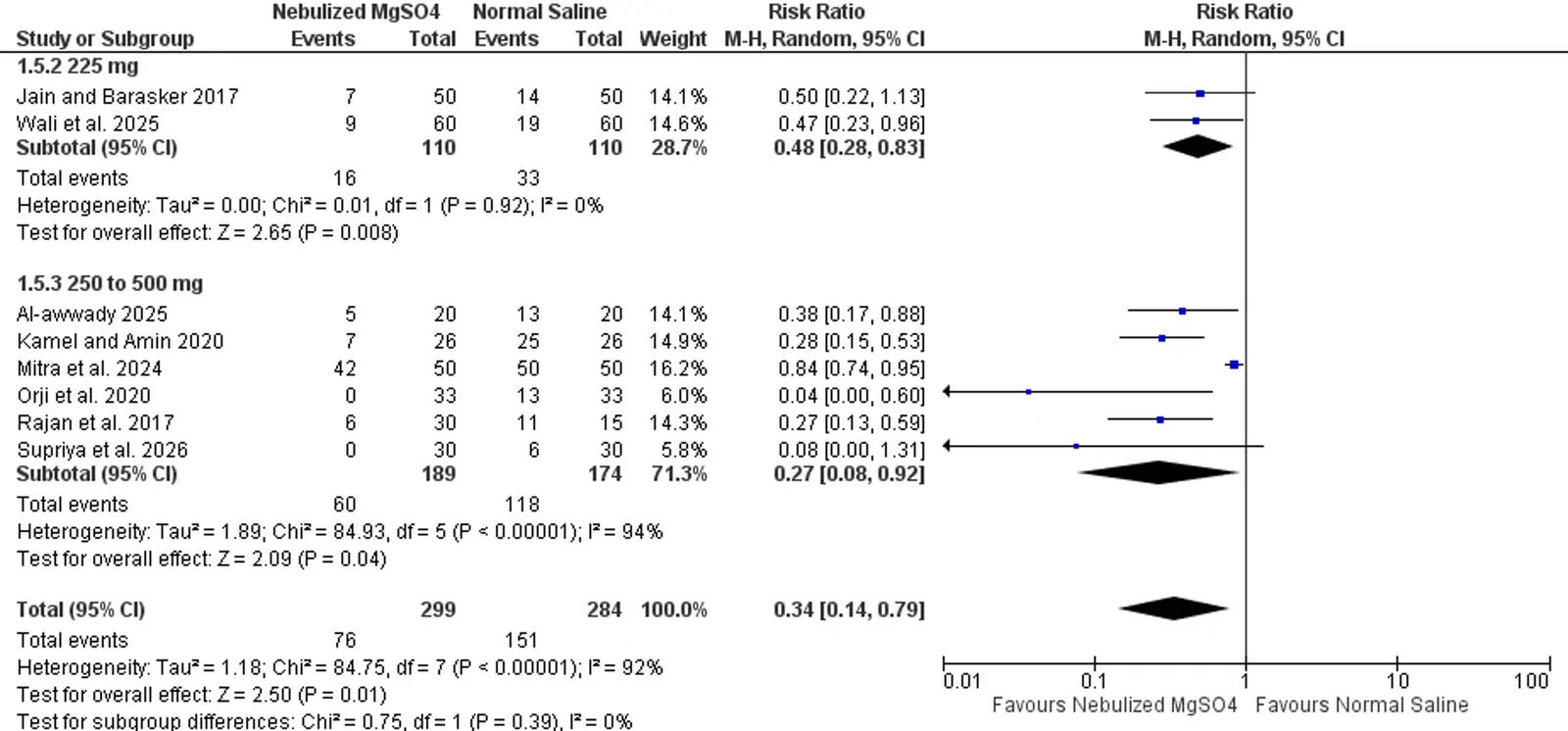
Post-operative sore throat (POST) at 12 hours (subgroup)

Eight studies comprising 583 patients (299 in the Nebulized MgSO₄ group and 284 in the normal saline group) investigated for POST at 12 h. The pooled analysis revealed significantly reduced incidence of POST at 12 h in patients receiving MgSO4 compared with normal saline (RR 0.34, 95% CI 0.14–0.79 ;*P* = 0.01; I²=92% ) (Fig. 10). Upon exclusion of the outlier study (Mitra et al. 2024), heterogeneity was effectively eliminated ( I² = 4% ), and the pooled result showed a highly significant benefit for Nebulized MgSO4 (RR = 0.34, 95% CI [0.24, 0.48], *p* < 0.00001). The detailed results of the sensitivity analysis are presented in Supplementary Fig. 3. Subgroup analysis was conducted on 7 studies (*n* = 483) and demonstrated a significant reduction in risk for both the 225 mg (RR = 0.48, 95% CI 0.28–0.83, *p* = 0.008 ) and 250 to 500 mg (RR = 0.27, 95% CI 0.08–0.92, *p* = 0.04) dosage groups, with no significant differences observed between the subgroups (Fig. 11).

#### Incidence of hoarseness at 4 hours

**Fig. 12:**
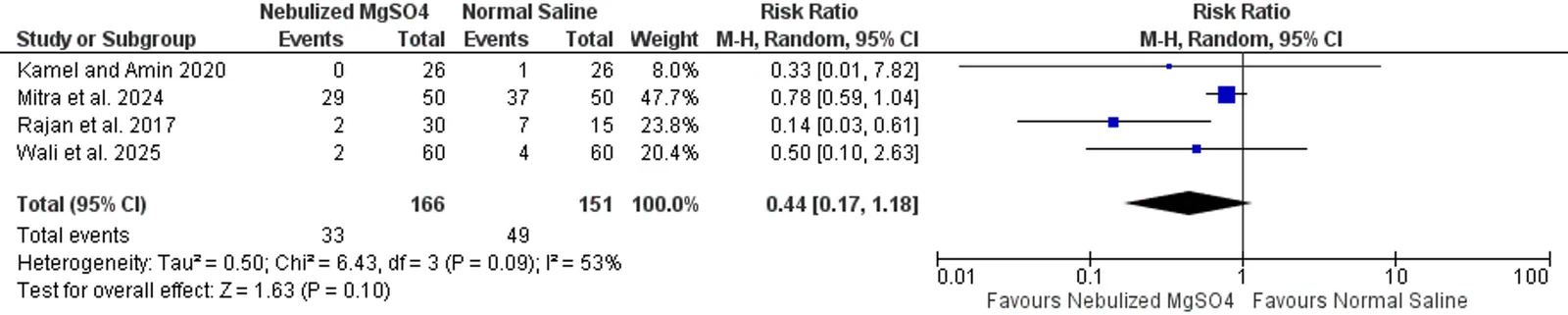
Incidence of hoarseness at 4 hours

**Fig. 13:**
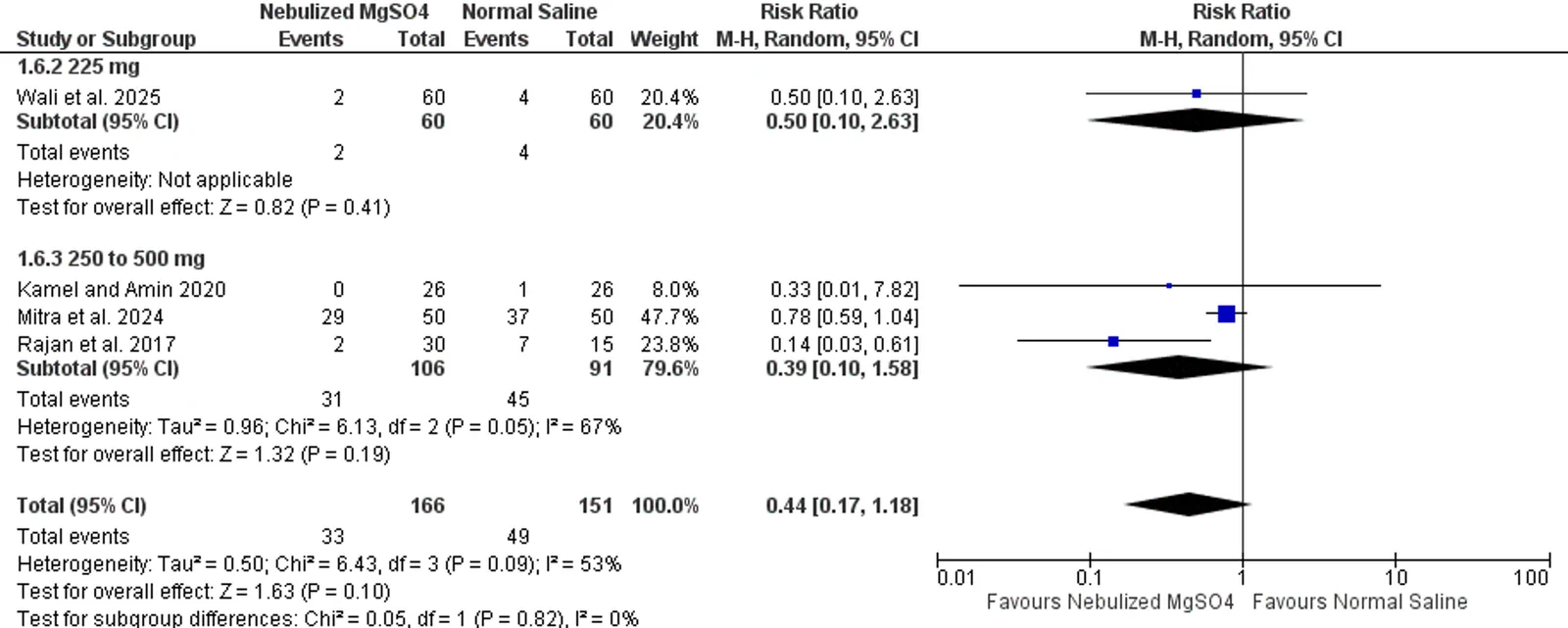
Incidence of hoarseness at 4 hours (subgroup)

Four studies comprising 317 patients (166 in the Nebulized MgSO₄ group and 151 in the normal saline group) investigated the incidence of hoarseness at 4 h. The pooled analysis did not show a statistically significant reduction in incidence of hoarseness at 4 h in patients receiving MgSO4 compared with normal saline (RR 0.44, 95% CI 0.17–1.18 ;*P* = 0.10; I²=53% ) (Fig. 12). Subgroup analysis for hoarseness at 4 h revealed no significant effect for either the 225 mg (RR = 0.50, 95% CI 0.10– 2.63, *p* = 0.41) or the 250–500 mg dosage (RR = 0.39, 95% CI 0.10–1.58, *p* = 0.19), with no significant variation between subgroups (Fig. 13).

#### Incidence of post-operative cough at 4 hours

**Fig. 14:**
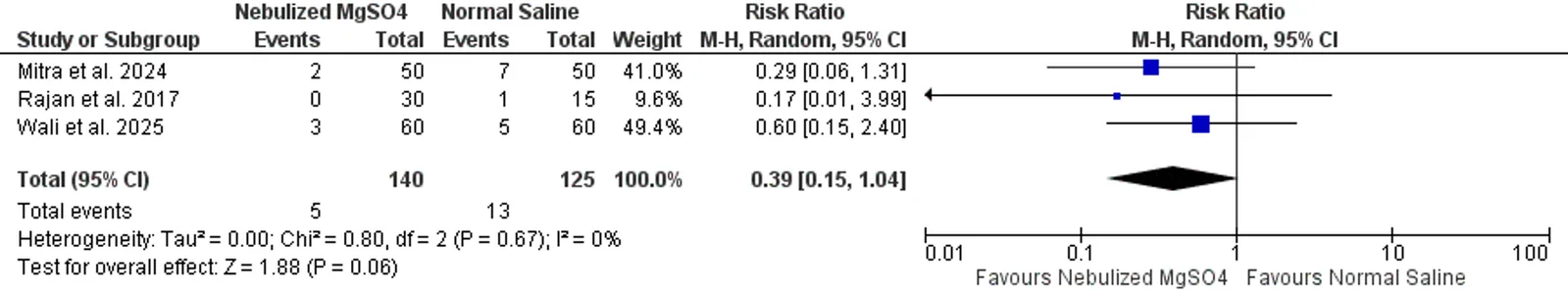
Incidence of post-operative cough at 4 hours

**Fig. 15:**
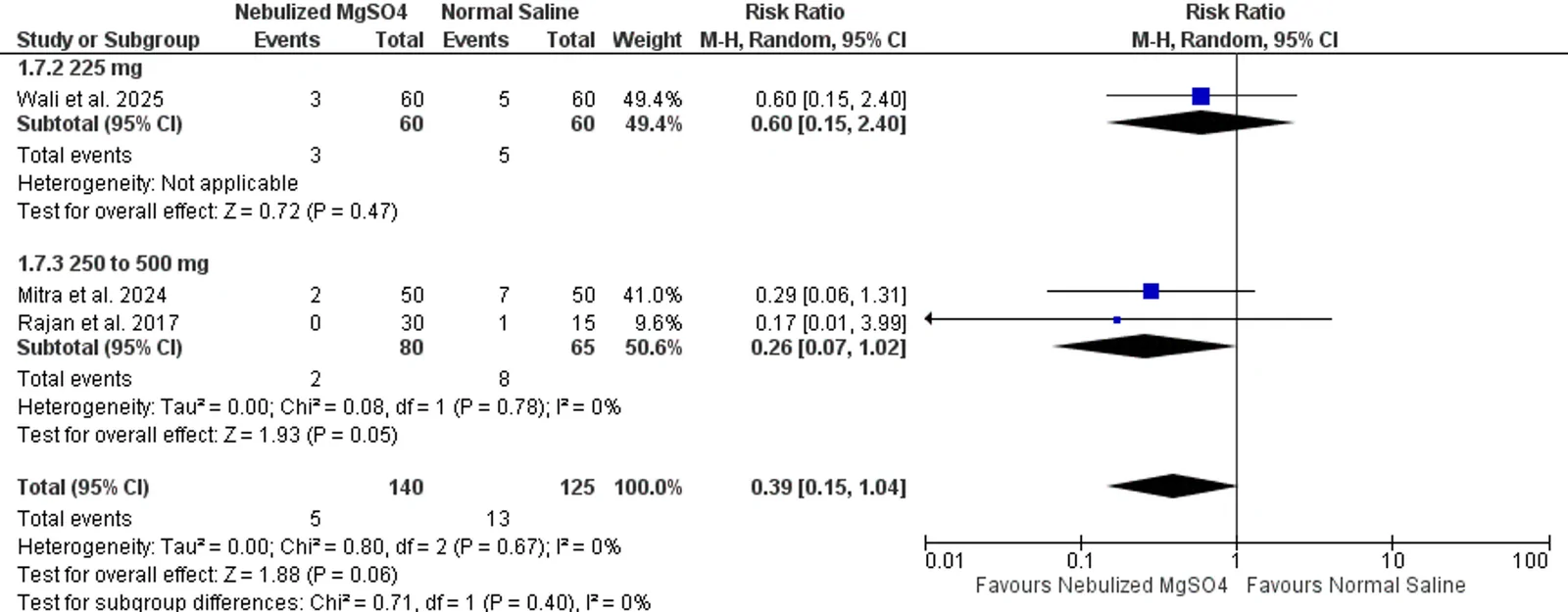
Incidence of post-operative cough at 4 hours (subgroup)

Three studies comprising 265 patients (140 in the Nebulized MgSO₄ group and 125 in the normal saline group) investigated the incidence of Post-operative Cough at 4 h. The pooled analysis revealed a trend toward reduced incidence, though it did not reach statistical significance of Post-operative Cough at 4 h in patients receiving MgSO4 compared with normal saline (RR 0.39, 95% CI 0.15 − 1.04 ;*P* = 0.06; I²=0% ) (Fig. 14). From subgroup analysis Neither the 225 mg (RR = 0.60, 95% CI 0.15–2.40 *p* = 0.47) nor the 250–500 mg (RR = 0.26, 95% CI 0.07– 1.02, *p* = 0.05) dosage groups reached statistical significance, and no significant differences were observed between these subgroups (Fig. 15).

#### Hypotension / hemodynamic instability

**Fig. 16:**
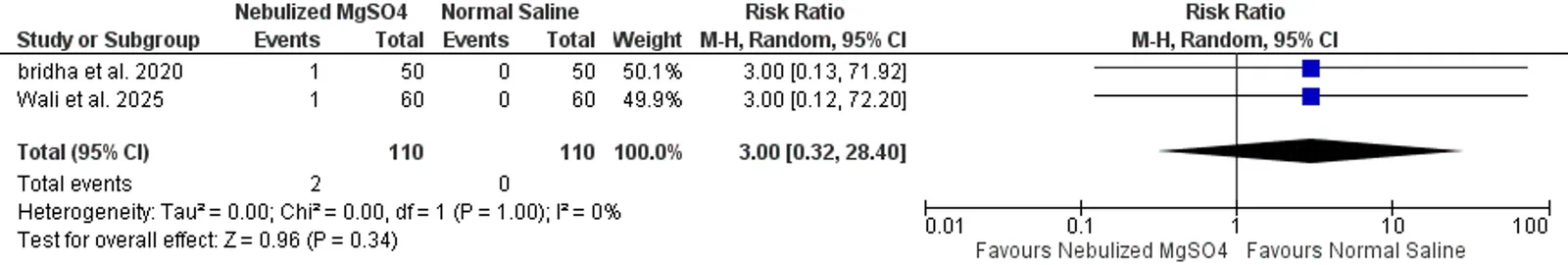
Hypotension / hemodynamic instability

Two studies comprising 220 patients (110 in the Nebulized MgSO₄ group and 110 in the normal saline group) investigated the incidence of hemodynamic instability. The pooled analysis revealed no significant hypotension in patients receiving MgSO4 compared with normal saline (RR 3, 95% CI 0.32–28.40 ;*P* = 0.34 ; I²=0% ) (Fig. 16). Subgroup analysis was not applicable.

### Publication bias

Assessment of publication bias was limited to the primary outcome (POST at 24 h). While visual inspection of the funnel plot demonstrated a generally symmetrical distribution, a critical limitation must be acknowledged: all twelve included trials reported uniform effect directions favoring magnesium sulfate. Given the relatively small sample sizes of these individual studies and their geographically restricted origins, this uniform positivity raises a high index of suspicion for small-study effects and regional publication bias. Because standard meta-analytical tools cannot definitively differentiate true homogeneity of effect from the systematic non-publication of negative trials, the risk of publication bias cannot be entirely excluded. The funnel plot is presented in Supplementary Fig. 13.

### Trial sequential analysis

The trial sequential analysis (TSA) revealed that for POST incidence evaluated at 2, 4, 8, 12, and 24 h, the cumulative Z-curves successfully breached both the trial sequential monitoring thresholds and the traditional significance boundaries, fulfilling the optimal information size requirements. This indicates that the protective effect of nebulized magnesium sulfate against POST is statistically robust and unlikely to be due to random error. In contrast, for the secondary outcomes of postoperative hoarseness, the cumulative Z-curves crossed the conventional significance boundaries and the trial sequential monitoring boundaries but did not reach the required optimal information sizes. For postoperative cough, the cumulative Z-curves crossed conventional significance boundaries only, while failing to reach the optimal information size. These findings suggest that the current evidence for these secondary outcomes remains inconclusive and underpowered. The detailed trial sequential analysis results are presented in Supplementary Figs. 6–12.

## Discussion

This meta-analysis evaluated the effectiveness of preoperative nebulized magnesium sulfate (MgSO₄) compared with placebo or normal saline for the prevention of postoperative sore throat (POST) in adult patients undergoing general anesthesia. Our primary analysis demonstrated that nebulized MgSO₄ was associated with a significant reduction in the incidence of POST at 24 h after extubation. This protective effect was also observed consistently during the early postoperative period, with significant reductions at 2, 4, 8, and 12 h. Subgroup analysis suggested that the 225 mg dose was comparable to higher cumulative doses (250–500 mg), indicating that increasing the dose within this range may not provide additional clinical benefit.

Regarding secondary outcomes, nebulized MgSO₄ showed a favorable trend toward reduced postoperative hoarseness and cough at 4 h; however, these findings did not reach statistical significance. Importantly, the intervention demonstrated a comparable safety profile, with no significant increase in hypotension or bradycardia compared with controls.

The reduction in POST at 24 h aligns with findings from a major systematic review by Singh et al. [14], which reported thatagnesium Sulfate decreased POST incidence. However, that review included heterogeneous routes of administration (intravenous, gargle, and topical), potentially introducing variability in systemic absorption and local effects. By restricting inclusion to the nebulized route, our analysis provides more focused evidence supporting the efficacy of localized airway administration. This is consistent with more recent randomized trials included in our review. For example, Supriya et al. 2026 demonstrated that nebulized magnesium reduced POST, although lignocaine was more effective in suppressing cough reflex [26]. Similarly, Al-awwady reported beneficial effects in patients undergoing thyroidectomy, supporting the role of topical airway therapy in specific surgical populations [22].

Moderate heterogeneity was observed in the primary analysis (I² = 62%). Sensitivity analysis identified Mitra et al. (2024) as the primary driver of heterogeneity. A critical appraisal of this trial reveals an unusually high baseline incidence of POST, with 100% of patients in the control arm (50/50) developing postoperative sore throat, an event rate significantly higher than the 40–60% average observed in the other included trials. This anomaly likely magnified their reported effect size. The inflated control event rate may be attributable to specific institutional protocols regarding endotracheal tube sizing, variations in objective cuff-pressure monitoring, or the use of a highly sensitive subjective POST assessment scale.

The temporal reduction in POST across early postoperative time points is biologically plausible. Nebulized magnesium is thought to stabilize mucosal membranes and inhibit calcium influx, thereby reducing the release of inflammatory mediators triggered by endotracheal tube cuff pressure [32]. The early postoperative period is clinically relevant, as airway discomfort is most pronounced during this phase. A 2025 study comparing magnesium spray versus gargle also supported the efficacy of local application with favorable compliance outcomes [33]. Subgroup findings indicating comparable efficacy between 225 mg and higher doses may have practical implications, as lower doses could potentially reduce theoretical risks of local irritation while maintaining benefit [34].

Regarding airway morbidity other than sore throat, our results showed no statistically significant reduction in the incidence of hoarseness or cough at 4 h, although a favorable trend was observed for both. However, this is in contrast to a study by Borazan et al., [35] which indicated that topical magnesium significantly reduced the incidence of hoarseness. This could be explained by our strict inclusion criteria of only nebulized studies, as opposed to other studies that may include a combination of interventions or lozenges. Additionally, the etiology of hoarseness is often multifactorial, including laryngeal trauma or edema, which may not be adequately alleviated by mucosal analgesia alone [8]. However, a comparative assessment of nebulized magnesium and lignocaine in 2026 did indicate that while magnesium was superior for sore throat, lignocaine was slightly more effective in suppressing cough reflexes, implying different underlying mechanisms [26]. The absence of statistical significance regarding cough in our study may also be due to the relative paucity of studies assessing this particular outcome, thereby diminishing our statistical power. Nevertheless, the trend observed is biologically valid, given magnesium’s known properties of relaxing bronchial smooth muscle and reducing airway reflexes [36].

Safety is always a major issue in magnesium therapy. Preliminary data from two trials (*n* = 220) in our meta-analysis suggests no increased risk of hemodynamic instability between the magnesium and control groups. However, because this outcome is supported by low-certainty evidence, the data is currently underpowered to draw definitive safety conclusions for high-risk cardiovascular patients. This supports the effectiveness of the nebulized technique in reducing systemic absorption, thus avoiding the vasodilation and myocardial depression that are commonly seen in intravenous magnesium therapy administration [37]. A recent randomized double-blind study in 2025 comparing intravenous and nebulized Magnesium Sulfate administration further supported this, showing that the nebulized technique offered a similar attenuation of the pressor response to intubation without the systemic side effects observed in the IV group [38]. The safety profile of nebulized magnesium makes it an ideal agent in ambulatory surgery and in patients with limited cardiovascular reserve who cannot tolerate the hemodynamic effects commonly seen with other adjuncts such as clonidine or intravenous lidocaine [39].

To assess the robustness of our findings and reduce the risk of type I error due to repetitive testing, Trial Sequential Analysis (TSA) was performed. For the primary outcome of POST at 2, 4, 8, 12, and 24 h, the cumulative Z-curves crossed the conventional monitoring boundaries and reached the optimal information size, suggesting that the available evidence is statistically robust for the prevention of POST with nebulized Magnesium Sulfate.

In contrast, TSA for secondary outcomes, including postoperative hoarseness and cough, demonstrated that the optimal information sizes have not yet been reached. Although conventional meta-analysis indicated favorable trends, the cumulative Z-curves crossed the trial sequential monitoring boundaries for postoperative hoarseness only, indicating that these findings remain potentially vulnerable to random error.

Taken together, the TSA findings strengthen confidence in the protective effect of nebulized Magnesium Sulfate for POST, while highlighting the need for additional adequately powered randomized controlled trials to clarify its impact on secondary airway morbidities.

### Strengths and limitations

This meta-analysis has a number of important strengths. Firstly, it was conducted solely within the nebulized route of administration, allowing for precise guidance that is free from the confounding influence of systemic or gargle formulations identified in other systematic reviews. Secondly, we conducted thorough sensitivity analyses to support the validity of our outcomes, ensuring that the results are statistically sound and not influenced by outliers. Thirdly, to the best of our knowledge, this meta-analysis represents the first to solely investigate the efficacy of the nebulized route of Magnesium Sulfate in POST prophylaxis. Moreover, the inclusion of recent randomized controlled trials until 2026 ensures that the meta-analysis is contemporary to current anesthetic practice, including current practice of endotracheal tube cuff management.

However, there are a number of limitations to our meta-analysis. A major limitation of this meta-analysis is the restricted external validity and geographic clustering of the evidence base. All included trials were conducted in lower-middle-income countries across South Asia, the Middle East, and Africa, with a complete absence of data from North American or European cohorts. Furthermore, there is a significant pharmacological barrier to global generalizability: there is currently no universally approved, commercially manufactured dedicated nebulizer solution of Magnesium Sulfate for POST prophylaxis. Consequently, the interventions in these trials rely on the off-label nebulization of intravenous Magnesium Sulfate formulations. In heavily regulated healthcare systems, hospital pharmacies may prohibit the routine off-label compounding of intravenous drugs for minor postoperative airway morbidities, rendering this intervention practically unavailable to many practitioners. Therefore, this meta-analysis should be interpreted as a proof-of-concept synthesis that highlights the clinical potential of the drug, establishing the foundational evidence required to justify large-scale, multi-center trials in diverse global populations to evaluate regulatory approval for a dedicated inhalational formulation. Furthermore, the uniform positivity and relatively small sample sizes of the included trials suggest a potential for publication bias and small-study effects that cannot be definitively ruled out, necessitating a cautious interpretation of the magnitude of the reported treatment effect. Finally, although the number of studies reporting secondary outcomes of cough and hoarseness was not small, it was not sufficient to allow for the detection of significant differences for these outcomes.

### Clinical implications

The results of this meta-analysis have significant implications for clinical practice in the perioperative setting. Sore throat is one of the most common causes of patient complaints after general anesthesia. The results of this meta-analysis support the use of nebulized Magnesium Sulfate as a simple, cost-effective, and non-invasive prophylactic strategy. The dose of 225 mg demonstrates efficacy comparable to higher doses, which may assist in the standardization of protocols without overstating formal non-inferiority. Since there are no hemodynamic side effects, this strategy can be offered to a wide range of patients undergoing surgery, including those with cardiovascular comorbidities.

## Conclusion

In conclusion, this meta-analysis demonstrates that preoperative nebulized Magnesium Sulfate is effective in reducing the incidence of sore throat 24 h post-operatively and in the early postoperative period compared to the placebo. The treatment is also safe, with no significant hemodynamic side effects, and can be administered at a lower dose. Although there was a trend towards a reduction in hoarseness and cough, it was not statistically significant. These results support the use of nebulized Magnesium Sulfate in multimodal prophylactic regimens for airway management.

## Supplementary Information

Supplementary Material 1.

## Data Availability

The data generated and/or analyzed during the current study are available from the corresponding author on reasonable request.
