# Supplementary material for: Nebulized magnesium sulfate reduces postoperative sore throat following general anesthesia: a systematic review, meta-analysis, and trial sequential analysis
Source: BMC Anesthesiol. 2026 Jul 6;26:581. doi: 10.1186/s12871-026-04066-6 (PMC13617930; doi:10.1186/s12871-026-04066-6)
Supplement: Supplementary file 1 [file 12871_2026_4066_MOESM1_ESM.docx]

| outcome | No of participants (studies) | Effect estimates (95% CI) | Risk of bias | Inconsistency | Indirectness | Imprecision | Publication bias | Overall quality |
| --- | --- | --- | --- | --- | --- | --- | --- | --- |
| Incidence of post operative sore throat (POST) at 24 hrs | 1023 (12) | RR=0.25 (0.14 -0.42) | Not serious | serious | Not serious | Not serious | Serious | Low |
| POST at 2 hrs | 663(9) | RR=0.43 (0.32-0.58) | Not serious | Not serious | Not serious | Not serious | Not applicable (NA) | High |
| POST at 4 hrs | 703(9) | RR =0.3 (0.19-0.77) | Not serious | Serious | Not serious | Not serious | NA | Moderate |
| POST at 8 hrs | 358(5) | RR=0.49 (0.23-1.01) | Not serious | Serious | Not serious | Serious | NA | Low |
| POST at 12 hrs | 583(8) | RR=0.34(0.14-0.79) | Not serious | Serious | Not serious | Not serious | NA | Moderate |
| Incidence of hoarseness at 4 hrs | 317(4) | RR=0.44 (0.17-1.18) | Not serious | Serious | Not serious | Serious | NA | Low |
| Incidence of post operative cough at 4 hrs | 265(3) | RR=0.39 (0.15-1.04) | Not serious | Not serious | Not serious | serious | NA | Moderate |
| Hypotention/Hemodynamic instability | 220(2) | RR=3 (0.32-28) | serious | Not serious | Not serious | serious | NA | Low |

**Table 1: Grade Assessment**

| Database | Search string | Results |
| --- | --- | --- |
| Pubmed | ("Magnesium Sulfate"[Mesh] OR "Magnesium Sulfate" OR "Magnesium Sulphate" OR MgSO4 OR Magnesium)  AND  ("Administration, Inhalation"[Mesh] OR "Nebulizers and Vaporizers"[Mesh] OR "Aerosols"[Mesh] OR Nebuliz* OR Nebulis* OR Aerosol* OR Inhal* OR Atomiz*)  AND  ("Pharyngitis"[Mesh] OR "Hoarseness"[Mesh] OR "Cough"[Mesh] OR "Intubation, Intratracheal"[Mesh] OR "Postoperative Complications"[Mesh] OR "Sore Throat" OR "Postoperative sore throat" OR POST OR Hoarseness OR Cough OR Intubation) | 132 |
| Embase | ("Magnesium Sulfate" OR "Magnesium Sulfate" OR "Magnesium Sulphate" OR "MgSO4" OR "Magnesium")  AND  ("Administration, Inhalation" OR "Nebulizers and Vaporizers" OR "Aerosols" OR "Nebuliz" OR "Nebulis" OR "Aerosol" OR "Inhal" OR Atomiz")  AND  ("Pharyngitis" OR "Hoarseness" OR "Cough" OR "Intubation, Intratracheal" OR "Postoperative Complications" OR "Sore Throat" OR "Postoperative sore throat" OR POST OR Hoarseness OR Cough OR Intubation) | 962 |
| Scopus | ("Magnesium Sulfate" OR "Magnesium Sulphate" OR "MgSO4" OR "Magnesium")  AND  ("Nebulizer" OR "Nebulizers" OR "Nebulization" OR "Nebulisation" OR "Inhalation" OR "Aerosol" OR "Aerosols" OR "Atomization" OR "Atomisation")  AND  ("Postoperative Sore Throat" OR "Sore Throat" OR "Hoarseness" OR "Cough" OR "Pharyngitis" OR "Intubation" OR "Endotracheal Intubation") | 191 |

**Table 2: Detailed search strategy**

Figure 1: Risk of bias

Figure 2: Post-Operative Sore Throat (POST) at 24 Hours (sensitivity)

Figure 3: Post-Operative Sore Throat (POST) at 12 Hours (sensitivity)

Figure 4: Post-Operative Sore Throat (POST) at 8 Hours (sensitivity)

Figure 5: Post-Operative Sore Throat (POST) at 4 Hours (sensitivity)

Figure 6: Trial sequential analysis of Post-Operative Sore Throat (POST) at 24 Hours

Figure 7: Trial sequential analysis of Post-Operative Sore Throat (POST) at 12 Hours

Figure 8: Trial sequential analysis of Post-Operative Sore Throat (POST) at 8 Hours

Figure 9: Trial sequential analysis of Post-Operative Sore Throat (POST) at 4 Hours

Figure 10: Trial sequential analysis of Post-Operative Sore Throat (POST) at 2 Hours

Figure 11: Trial sequential analysis of Post-Operative Sore hoarseness at 4 Hours

Figure 12: Trial sequential analysis of Post-Operative Cough at 4 Hours

Figure 13: Funnel plot of Post-Operative Sore Throat (POST) at 24 Hours
